# The Role of Abiotic Environmental Conditions and Herbivory in Shaping Bacterial Community Composition in Floral Nectar

**DOI:** 10.1371/journal.pone.0099107

**Published:** 2014-06-12

**Authors:** Michal Samuni-Blank, Ido Izhaki, Sivan Laviad, Avi Bar-Massada, Yoram Gerchman, Malka Halpern

**Affiliations:** 1 Department of Evolutionary and Environmental Biology, Faculty of Natural Sciences, University of Haifa, Mount Carmel, Haifa, Israel; 2 Department of Biology and Environment, University of Haifa at Oranim, Oranim, Tivon, Israel; University Paris South, France

## Abstract

Identifying the processes that drive community assembly has long been a central theme in ecology. For microorganisms, a traditional prevailing hypothesis states that “everything is everywhere, but the environment selects”. Although the bacterial community in floral nectar may be affected by both atmosphere (air-borne bacteria) and animals as dispersal vectors, the environmental and geographic factors that shape microbial communities in floral nectar are unknown. We studied culturable bacterial communities in *Asphodelus aestivus* floral nectar and in its typical herbivorous bug *Capsodes infuscatus*, along an aridity gradient. Bacteria were sampled from floral nectar and bugs at four sites, spanning a geographical range of 200 km from Mediterranean to semi-arid conditions, under open and bagged flower treatments. In agreement with the niche assembly hypothesis, the differences in bacterial community compositions were explained by differences in abiotic environmental conditions. These results suggest that microbial model systems are useful for addressing macro-ecological questions. In addition, similar bacterial communities were found in the nectar and on the surface of the bugs that were documented visiting the flowers. These similarities imply that floral nectar bacteria dispersal is shaped not only by air borne bacteria and nectar consumers as previously reported, but also by visiting vectors like the mirid bugs.

## Introduction

Identifying processes that drive community assembly have long been a central theme in ecology [1], [2]. In general, there are two distinct views of community structuring: niche assembly [3] and dispersal assembly [4], [5]. Dispersal assembly (which was the foundation for MacArthur and Wilson's theory of island biogeography [4], and Hubbell's neutral theory [5]), assumes that communities comprise an assembly of species that arrive by chance dispersal events, regardless of differences in niche requirements among them. A central prediction of the dispersal assembly hypothesis is that community similarity decreases with increasing geographic distance, regardless of differences in environmental variables among sites [5]. By contrast, niche assembly suggests that species-specific life-history traits and environmental adaptations explain the abundance and distribution of species, their coexistence and biodiversity [6]–[8]. It thus predicts that communities located at sites with similar environmental conditions will have similar species compositions [9], [10]. Hence, the spatial patterns of species assemblages will be affected by environmental differences among sites. Our ability to disentangle the effects of environmental and spatial factors on community composition is vital in understanding the determinants of species richness, variation and biodiversity patterns [11], [12].

Until recently, most of our knowledge of the mechanisms that govern species composition and diversity emerged from studies focusing on macro-organisms such as plants and animals [13]–[14], whereas factors which shape microbial communities have only recently been addressed. The traditional hypothesis about the distribution of microorganisms is that “everything is everywhere, but the environment selects” [15]. The rationale behind this is that microorganisms are extremely abundant, proliferate rapidly and disperse easily. Hence, it is expected that dispersal limitation has no impact on bacterial community composition. However, a review of published studies revealed that the importance of local environmental factors for bacterial community composition has been much more intensively studied than the importance of regional factors, such as dispersal. Furthermore, only few attempts have been made to evaluate simultaneously the relative importance of the two types of factors for bacterial community composition [16]. This is rather surprising, because not only do microorganisms constitute the majority of species, individuals and biomass in many ecosystems, they also play key roles in community and ecosystem function [17]–[20].

One possible mechanism of bacterial dispersal among plant hosts across space is transfer by animal vectors. Most plant species on earth are animal-pollinated [21]. Floral nectar is regarded as the most important calorific and nutritional reward which animal-pollinated plants furnish to attract pollinators [22]–[24]. Nectar chemistry serves also as protection from nectar robbers and nectar infecting microorganism [25]–[27]. However, yeast and bacteria have been found to inhabit floral nectar [28]–[31]. These microorganisms may influence the chemistry of the nectar, therefore also impact plant-pollinator interactions [31], [32]. Microorganisms are transferred across flowers and sites not only by wind or precipitation but also by various nectar consumers such as insects and birds [32]–[35]. Indeed, a recent study demonstrated that honeybees may introduce bacteria into the nectar and/or may be contaminated by bacteria introduced into the nectar by other sources [31].

In this study, we examined the effects of the plants' environmental conditions across sites and herbivory on the nectar bacterial community composition in floral nectar. To that end, we studied the composition of communities of culturable bacteria in floral nectar of *Asphodelus aestivus* along a 200 km climatic gradient in Israel. The plants' environment may affect nectar production rates, sugar content and chemistry by mechanisms mediated via the plant [36]. So, a climatic gradient may have a significant effect on shaping the nectar which may in turn affect bacterial communities' composition. We also studied the culturable bacterial communities associated with *Capsodes infuscatus* bugs attacking *A. aestivus*. By manipulating the access of nectar consumers to the flower, we constructed a unique experimental system that revealed the role of a potential insect vector versus airborne-origin bacterial sources, in shaping the composition of bacterial communities in floral nectar.

## Materials and Methods

### Ethics Statement

No specific permission was required for plant samplings since the field studies did not involve endangered or protected species. The exact location of sampling points for this study is indicated in Table 1.

**Table 1:** Environmental factors at each of the four sites (Goral, Nadiv, Bashan and Golan).

Site	Location	Elevation (meters above sea level)	Temperature (°C)	Annual precipitation (mm)	Soil
Goral	31.36721N 34.83212E	395	13.2	297	Brown lithosols and loessial arid brown soils
Nadiv	32.55565N 34.94828E	125	13.8	569	Terra rossas, brown rendzinas and pale rendzinas
Bashan	33.01230N 35.82997E	830	12	633	Basaltic brown Mediterranean soils and basaltic lithosols
Golan	33.10816N 35.77085E	900	9.3	832	Basaltic brown Mediterranean soils and basaltic lithosols

For temperature we used average data of the month sampled.

### Plant Natural History

**Figure 1:**
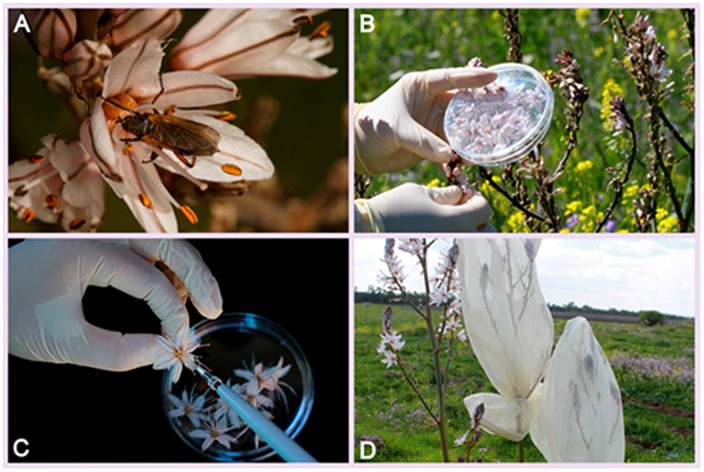
*Asphodelus aestivus*. A, Flower and a consumer fly (*Eempidoidea*); B, Flower collection; C, Nectar collection; D, Bagged inflorescences.

*Asphodelus aestivus*, also known as *Asphodelus ramosus* [*Liliaceae*] (Fig. 1A) is a common Mediterranean geophyte. The plant produces from tens to hundreds of flowers on each paniculate inflorescence. The population typically consists of long-lived, distinct and dense clonal patches of plants. Individual flowers last between 24 h and 48 h depending on temperature; on days colder than 13 °C they last longer [37]. The main pollinators of *A. aestivus* are *Hymenoptera*, however, other insects were also documented visiting the flowers (Fig. 1A) [37], [38]. According to Samocha and Sternberg [39] the plants' environmental conditions (time of day, temperature and relative humidity) do not affect nectar sugar concentration, which was found to be ∼60% [39].

### Bugs Natural History

*Capsodes infuscatus* bugs (*Hemiptera: Miridae*) attack *Asphodelus aestivus* with their piercing-sucking mouthparts. These mirid bugs feed on *A. aestivus* leaf cells, apical meristem of new inflorescences, buds, flowers and fruits [40]. Developing inflorescence stalks are their preferable food source [39]. This bug can suppress the development of the inflorescence and significantly reduce nectar and fruit production [39]–[41].

### Study Sites

**Figure 2:**
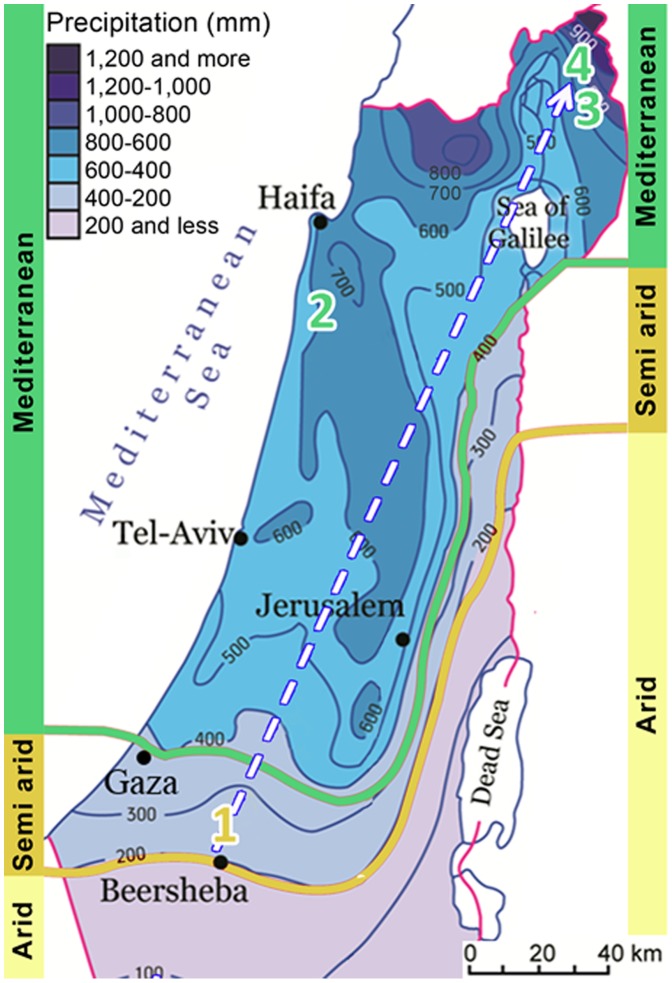
Study sites along the climatic gradient (white arrow). 1, Goral; 2, Nadiv; 3, Bashan; and 4, Golan. For more details see Table 1. The map was modified from: Israel: People and place (2007), with permission from Fein et al. (Fein Z, Segev M, Lavi R 2007, Israel: People and place. Cartography, Soffer R, Center for Educational Technology, Israel).

We utilized the unique climatic and ecological gradient which occurs in Israel from Semi-arid to Mediterranean ecosystems from south to north (arrow in Fig. 2). We allocated four sites (‘Goral’, ‘Nadiv’, ‘Bashan’ and ‘Golan’; numbered 1–4 in Fig. 2) along this gradient in areas with elevation from 125 to 900 m (Table 1). *Asphodelus aestivus* inhabits each of these sites which are also characterized by different temperature, precipitation, soil type, and elevation (Table 1).

### Nectar and bug sampling and bacteria culturing

We collected floral nectar from *Asphodelus aestivus* in the flowering season, from February to March 2013. At each site, we located plants within a radius of up to 200 m. We measured the location of each plant using an accurate GPS receiver (compass plus) integrated in a mobile phone. All sampled plants were distinct individuals, separated by at least 2 m [39]. Collection sessions at each site started at 8 am and lasted approximately five hours.

Sugar in floral nectar was analyzed by means of a hand-held refractometer (model BR62, Today's instruments Co. Ltd. Taipei Hsien, Taiwan). The mean (± SE) sugar equivalent concentration was 33.3%±1.5% (n = 40 flowers).

Open flowers (hereinafter 'open') from different plants (6.5±1.5 different plants per site, 7.4±0.3 flowers per plant), were aseptically sampled (Fig. 1B). Directly after flower collection, nectar was sampled by means of a plastic tip (Fig. 1C), and immediately spread onto R2A agar supplemented with 30% sucrose (HiMedia, Mumbai, India). Adding sucrose mimics the osmotic properties of the nectar. From each *A. aestivus* individual, we also aseptically collected *Capsodes infuscatus* adults (5.3±0.9 bugs per site). Each mired bug was collected from a distinct individual of *A. aestivus*, separated by at least 2 m [39]. Bugs were transferred immediately to a sterile tube and kept alive for a few hours at 4°C until use. After collecting the nectar and bugs we removed all the remaining open flowers and covered each individual with 1-mm-diameter netting ('bagged' treatment) to exclude nectar consumers and visitors (Fig. 1D). The removal of the remaining flowers might have induced a stress response in plants that might impact nectar microbial communities and, consequently bias the second sampling. But, this was the only way to ensure that only closed flowers would be sampled in the second round. We repeated nectar collection using the same methods 48 h after bagging the plants.

The dislodgement of bacteria from the surface of the bugs ('out') was performed by sonication according to Aizenberg-Gershtein et al. [31]. Samples were spread onto R2A agar (Himedia) supplemented with 30% sucrose (high osmotic pressure, as we focused on the identification of bacterial species that may also thrive in nectar). To isolate bacteria from within the bugs' bodies ('in'), bugs were transferred after sonication to a new sterile Eppendorf tube, homogenized with 200 ml of enzymatic lysis buffer [20 mM Tris-HCI (pH 8.0), 2 mM EDTA and 1.2% Triton-X-100], and spread onto R2A agar supplemented with 30% sucrose.

As nectar inoculation on agar plates was performed in the field, plates were kept at room temperature (25 °C) for up to seven hours until they reached the laboratory where they were incubated at 30 °C in the dark. Bug cultures were kept at 30 °C. Forty-eight hours after incubation, few colonies of each phenotypically distinct microbial type (e.g. colony size, color, transparency, texture) were picked and streaked on an R2A plate containing 20% sucrose to yield axenic cultures. In cases where only few colonies grew on a plate (10 or less) all the colonies where picked and streaked as described. This action was repeated four times for each clone. To avoid mixed colonies, these were also streaked on Luria–Bertani (LB) agar, at least once. All bacterial isolates were kept in LB with 30% glycerol (−80°C). Representative isolates of each morphotype were identified by amplifying and sequencing a 1501-bp internal fragment of the 16S rRNA gene using 11F and 1512R primers as described in Senderovich et al. [42]. The isolates were identified by means of the EzTaxon-e server (http://eztaxon-e.ezbiocloud.net/) [43] on the basis of 16S rRNA sequence data. Operational taxonomic units (OTUs) were defined at the level of 98% sequences similarities to the closest relatives. The sequences were submitted to the GenBank database under accession numbers KF436511 - KF436671 for bacteria found in nectar and KF436672 - KF436786 for bacteria found in bugs.

### Statistical analyses

We used rarefaction analysis to estimate the total expected culturable bacterial species richness in floral nectar (bagged and open flowers) and on bugs (in and out treatments), using the average of three nonparametric estimators for presence/absence data: Chao2, Jackknife1 and Incidence-Based Coverage Estimator of species richness (ICE) based on 100 randomizations without replacement. Nonparametric estimators for species richness present the least biased option, but because no particular estimator has consistently performed best [44], we averaged these three estimators. The estimated (rarefaction) species accumulation curves were calculated and plotted by the software EstimateS version 7.52 [45].

To visualize the difference among bacterial communities at the different sites and with different treatments, we performed a cluster analysis using R [46] with complete linkages based on the Bray-Curtis dissimilarity metric. Since the number of sites was insufficient to analyze the community–environment relationship using Mantel tests or generalized dissimilarity models [47], we quantified the variation in bacterial community compositions across sites (while accounting for the two treatments) and across treatments (while accounting for stratification in locations), using analysis of variance of distance matrices (Adonis) [48] which is a nonparametric version of multivariate analysis of variance (MANOVA). This analysis was also performed in R [46]. Finally, we used CANOCO version 4.5 [49] to perform Canonical Correspondence Analysis (CCA) to analyze the distribution of bacterial species accounting for temperature, elevation and precipitation gradients (Table 1) based on presence/absence data. In all cases, we set significance level at *P*<0.05.

## Results

### Nectar sampling

**Figure 3:**
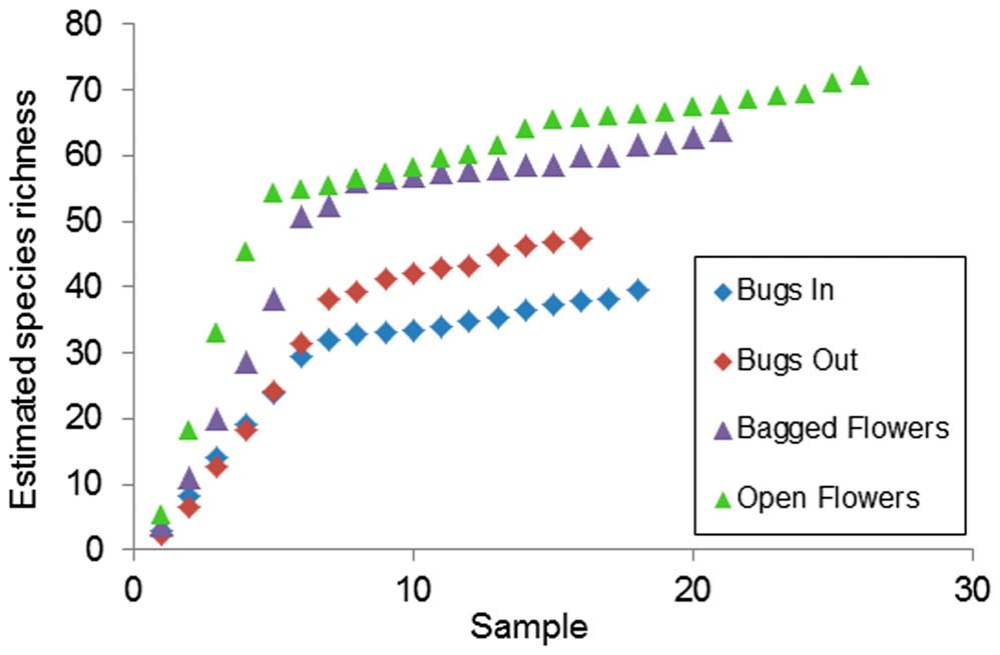
Estimated species richness (calculated as average of Chao2, Jackknife1 and ICE) in floral nectar of *Asphodelus aestivus* (open and bagged flowers) and in and out the mirid bug *Capsodes infuscatus*. The curves of the expected species richness approximately reach an asymptote, demonstrating that only a few more species would have been collected had the sampling effort been further increased.

A total of 161 representative isolates recovered from 52 nectar samples and from 28 distinct individual plants, were identified by amplifying and sequencing the 16S rRNA gene (Table 2). The mean observed number (± SE) of the operational taxonomic units (OTUs) per nectar sample, per site in open and bagged flowers was 13.5±2.5 and 10.8±3.0, respectively. Total estimated culturable bacterial species richness in floral nectar of all bagged and open flowers was 64.0 and 72.4, respectively (Fig. 3). Representatives of the *Bacilli*, *Gammaproteobacteria*, *Actinobacteria* and *Alphaproteobacteria* classes were found to account 46.5%, 24%, 23.5% and 6% of the nectar isolates, respectively (Table 2). Representatives of the *Alphaproteobacteria* were identified only from Nadiv and Golan sites. However, OTUs from the genus *Gluconobacter* were identified only from Golan (the northern-most sampled location). *Bacillus* (34%) and *Pseudomonas* (18%) (*Gammaproteobacteria*) were the two most common bacterial genera recovered; both were found in all the examined sites.

**Table 2:** List of bacterial isolates from nectar of *Asphodelus aestivus* sampled from the four sites (Goral, Nadiv, Bashan and Golan) and within each treatment (open or bagged).

	Goral		Nadiv		Bashan		Golan	
Closest relative in GenBank database	Open	Bagged	Open	Bagged	Open	Bagged	Open	Bagged
***Actinobacteria***								
*Agromyces salentinus*						1 (99.9)		
*Arthrobacter humicola*							2 (99.2)	3 (99.3)
*Arthrobacter nitroguajacolicus*		2 (99.5,99.8)						
*Arthrobacter oryzae*		1 (99.4)			1 (99.9)	1 (99.9)		
*Arthrobacter oxydans*	1 (100)	2 (99.9,100)			2 (100)			
*Arthrobacter pascens*		1 (99.6)				1 (99.7)	1 (99.5)	1 (99.4)
*Arthrobacter phenanthrenivorans*						1 (98.7)		1 (99.5)
*Brevibacterium frigoritolerans*						2 (100)		
*Curtobacterium flaccumfaciens*	1 (100)				2 (99.9–100)			5 (99.0–100)
*Microbacterium foliorum*						1 (99.3)		
***Bacilli***								
*Bacillus aerophilus*					1 (100)	1 (100)	1 (100)	
*Bacillus anthracis*		1 (98.6)						
*Bacillus aryabhattai*	1 (100)	1 (100)					5 (99.8–100)	1 (99.9)
*Bacillus endophyticus*					1 (99.7)			
*Bacillus flexus*					1 (100)			
*Bacillus licheniformis*	1 (99.7)							
*Bacillus megaterium*	1 (99.7)					1 (99.7)		
*Bacillus mojavensis*	1 (100)							
*Bacillus nealsonii*		1 (99.0)		1 (99.2)				
*Bacillus niacini*		1 (99.8)						
*Bacillus safensis*	3 (99.8–100)						1 (99.9)	
*Bacillus simplex*	2 (100)	1 (100)			1 (100)	1 (100)	4 (100)	6 (99.6–100)
*Bacillus sonorensis*	1 (99.0)							
*Bacillus subtilis subsp. inaquosorum*	1 (99.9)			1 (99.9)				
*Bacillus tequilensis*	7 (99.9)		2 (99.9,100)	2 (99.9)				1 (99.9)
*Brevibacillus agri*							2 (98.7,99.4)	1 (99.4)
*Fictibacillus nanhaiensis*		1 (99.5)						
*Leuconostoc holzapfelii*					1 (99.9)			
*Lysinibacillus sinduriensis*						1 (99.1)		
*Scopulibacillus darangshiensis*	1 (95.0)							
*Staphylococcus arlettae*	1 (100)							
*Staphylococcus cohnii subsp. cohnii*					1 (100)			
*Staphylococcus cohnii subsp. urealyticus*		3 (100)						
*Staphylococcus epidermidis*					1(100)			
*Staphylococcus hominis subsp. hominis*						2 (99.7,99.9)		
*Staphylococcus warneri*		1 (100)			1 (100)		1 (100)	
*Terribacillus saccharophilus*						1 (100)		1 (100)
***Alphaproteobacteria***								
*Gluconobacter kondonii*							4 (98.0–98.4)	
*Gluconobacter morbifer*							2 (97.2–98.0)	
*Gluconobacter sphaericus*							1 (98.4)	
*Neokomagataea tanensis*			2 (100)					
***Gammaproteobacteria***								
*Acinetobacter boissieri*			1 (100)					
*Acinetobacter nectaris*			4 (99.8)					
*Erwinia persicina*							2 (99.7–99.8)	
*Erwinia toletana*	2 (99.7)	1 (99.7)						
*Flavimonas oryzihabitans*	1 (99.2)							
*Lonsdalea quercina*							1 (99.9)	
*Pantoea eucalypti*							1 (99.8)	
*Pseudomonas azotoformans*							2 (99.9)	
*Pseudomonas baetica*					1 (99.3)			
*Pseudomonas cedrina subsp. fulgida*						2 (100)		
*Pseudomonas congelans*							1 (100)	
*Pseudomonas graminis*		1 (99.4)						
*Pseudomonas koreensis*		1 (100)						
*Pseudomonas lini*					10 (99.4–100)			
*Pseudomonas lutea*		1 (100)						
*Pseudomonas mohnii*		1 (99.6)						
*Pseudomonas syringae*					7 (99.2–99.8)			
*Pseudomonas viridiflava*			1 (100)				1 (99.6)	
*Rosenbergiella nectarea*	1 (99.5)		1 (98.9)					
**Total**	**26**	**21**	**11**	**4**	**31**	**16**	**32**	**20**

The numbers indicated in the table are the number of isolates and the numbers in parentheses are the percentage of 16S rRNA gene similarities to the closest known species. The sequences coverage of most isolates was 700–800 bp. For more details see Table S1.

**Figure 4:**
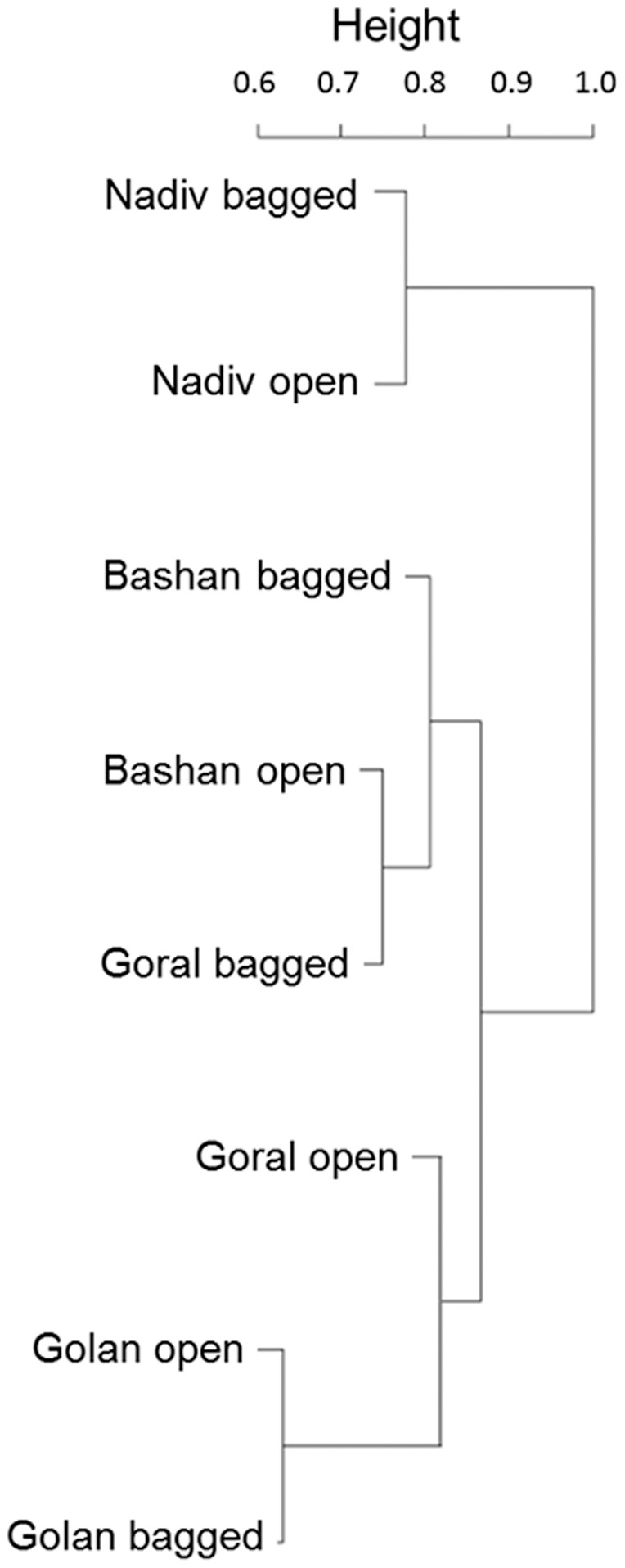
Nectar bacterial community composition clustered by site (Goral, Nadiv, Bashan and Golan) and treatments (open and bagged). Sites varied significantly in OTUs composition (Adonis test; *F*_3_ = 1.36, R^2^ = 0.5, *P*<0.05; Table 1).

**Figure 5:**
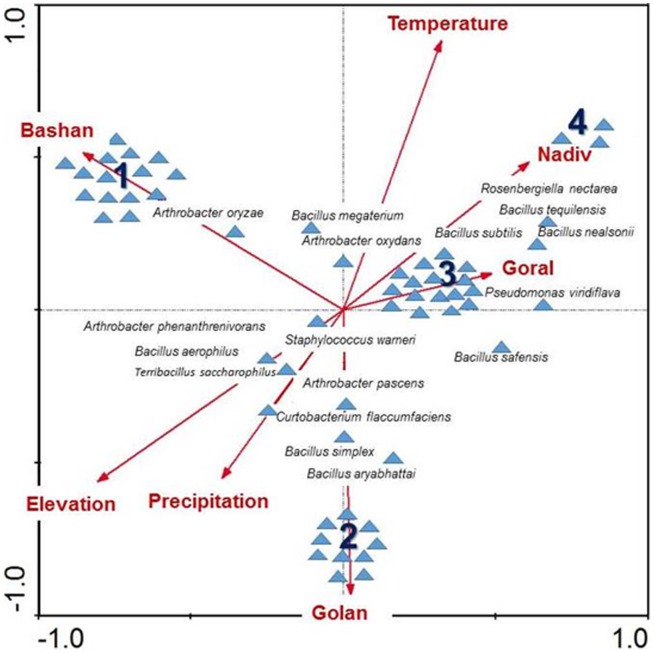
Variation of bacterial species isolates among the four different sites (Goral, Nadiv, Bashan and Golan) as shown by the ordination diagram (CCA). The distribution of bacterial species along the ordinates was not random (Monte Carlo test; *F* = 1.41, *P*<0.005) and thus can be explained by their different locations along the climatic gradient. The first two ordination axes explained 74.1% of the variance of species-environment relation. Identity of the species related to a single site is as follows: Group 1: *Agromyces salentinus*, *Leuconostoc holzapfelii, Pseudomonas lini, Arthrobacter chlorophenolicus, Lysinibacillus sinduriensis, Pseudomonas syringae, Bacillus endophyticus, Microbacterium foliorum, Staphylococcus cohnii subsp. cohnii, Bacillus flexus, Pseudomonas baetica, Staphylococcus epidermidis, Brevibacterium frigoritolerans, Pseudomonas cedrina subsp. fulgida, Staphylococcus hominis subsp. hominis*. Group 2: *Lonsdalea quercina, Arthrobacter humicola, Pantoea eucalypti, Brevibacillus agri, Pseudomonas azotoformans, Erwinia persicina, Pseudomonas congelans, Gluconobacter kondonii, Gluconobacter morbifer, Gluconobacter sphaericus*. Group 3: *Arthrobacter nitroguajacolicus, Bacillus anthracis, Bacillus licheniformis, Bacillus mojavensis, Bacillus niacin, Bacillus sonorensis, Erwinia toletana, Fictibacillus nanhaiensis, Flavimonas oryzihabitans, Pseudomonas graminis, Pseudomonas koreensis, Pseudomonas lutea, Pseudomonas mohnii, Scopulibacillus darangshiensis, Staphylococcus arlettae, Staphylococcus cohnii subsp. urealyticus*. Group 4: *Acinetobacter boissieri, Acinetobacter nectaris, Neokomagataea tanensis*.

Cluster analysis revealed that OTUs differed across sites, and that in all cases except Goral community similarity across treatments (open versus bagged), community similarity within sites was greater than community similarity across sites (Fig. 4). There was significant variation in OTUs' composition among sites (Adonis test; *F*_3_ = 1.36, *P*<0.05; Table 2). There was no significant variation in OTUs composition under open and bagged treatment (Adonis test; *F*_1_ = 1.07, NS). Canonical Correspondence Analysis (CCA) revealed that bacterial community composition differed along environmental gradients in temperature, precipitation and elevation and that different bacteria species were associated with different sites and environmental conditions (Fig. 5; Table 2). The distribution of bacterial species along the ordinates was not random (Monte Carlo test; *F* = 1.41, *P*<0.005).

### Bug sampling

A total of 115 representative isolates recovered from 34 mirid bug samples collected from 21 distinct individual plants, were identified based on 16S rRNA gene sequences (Table 3). The estimated bacterial species richness in bug ‘in’ and ‘out’ treatments was 39.5 and 47.3, respectively, which is <65% of what was found in floral nectar (Fig. 3). Representatives of the *Bacilli*, *Gammaproteobacteria*, *Actinobacteria* and *Alphaproteobacteria* classes accounted for 48.7%, 45.2%, 5.2% and 0.9% of the bug's bacterial isolates, respectively (Table 3). Representatives of the *Actinobacteria* and *Alphaproteobacteria* were identified mostly inside the bugs. *Bacillus* (43%) and *Pantoea* (17%) were the two most common bacterial genera recovered; both were found in the in and out treatments. However, OTUs from the genus *Pantoea* were identified only at Nadiv.

**Table 3:** List of bacterial isolates from *Capsodes infuscatus* at the four sites (Goral, Nadiv, Bashan and Golan) and within each treatment (in or out).

Closest relative in GenBank database	Goral		Nadiv		Bashan	Golan	
	In	Out	In	Out	Out	In	Out
***Actinobacteria***							
*Arthrobacter oxydans*						1 (99.3)	
*Curtobacterium flaccumfaciens*			1 (100)				1 (100)
*Microbacterium foliorum*	2 (99.2–99.3)						
***Bacilli***							
*Bacillus aerophilus*				1 (100)		26 (99.6–100)	
*Bacillus anthracis*						2 (100)	
*Bacillus aryabhattai*		1 (100)				1 (100)	
*Bacillus cereus*						3 (99.9)	
*Bacillus endophyticus*					2 (99.7)		
*Bacillus flexus*					1 (100)		
*Bacillus licheniformis*				1 (99.8)			1 (96.7)
*Bacillus nealsonii*				1 (99.3)			
*Bacillus safensis*					1 (100)		
*Bacillus simplex*							2 (100)
*Bacillus subtilis subsp. inaquosorum*	1 (100)						
*Bacillus tequilensis*	2 (100)	2 (99.9)	1 (100)	1 (99.9)			
*Brevibacillus agri*					1 (99.4)	1 (99.6)	
*Staphylococcus saprphyticus*						1 (99.9)	2 (99.9)
*Staphylococcus warneri*			1 (99.0)				
***Alphaproteobacteria***							
*Gluconobacter morbifer*						1 (98.1)	
***Gammaproteobacteria***							
*Acinetobacter schindleri*	1 (99.9)						
*Erwinia persicina*			1 (99.2)	3 (99.3–99.9)			
*Erwinia toletana*			2 (96.1)	3 (94.9–95.7)			
*Flavimonas oryzihabitans*				4 (99.1)			
*Pantoea agglomerans*			3(99.4–99.7)	4 (99.4–99.8)			
*Pantoea brenneri*			7 (99.6–100)	3 (99.7–100)			
*Pantoea conspicua*			2 (95.0–95.4)	1 (94.9)			
*Pseudomonas costantinii*				1 (99.4)			
*Pseudomonas orientalis*						1 (100)	4 (99.8–100)
*Pseudomonas plecoglossicida*			1 (99.3)				
*Yersinia kristensenii*						10 (98.8–99.3)	2 (99.3)
**Total**	**6**	**3**	19	**2**3	**5**	**47**	**12**

The numbers indicated in the table are the number of isolates and the numbers in parentheses are the percentage of 16S rRNA gene similarities to the closest known species. The sequences coverage of most isolates was 700–850 bp. For more details see Table S2.

There was significant variation in OTUs composition between the in and open treatments (Adonis test; *F*_1_ = 1.87, *P*<0.05) and between the in and bagged treatments (Adonis test; *F*_1_ = 1.71, *P*<0.01). There was no significant variation in OTUs composition between the in and out treatments (Adonis test; *F*_1_ = 0.59, NS), between out and open treatments (Adonis test; *F*_1_ = 1.22, NS) and between out and bagged treatments (Adonis test; *F*_1_ = 1.18, NS).

**Figure 6:**
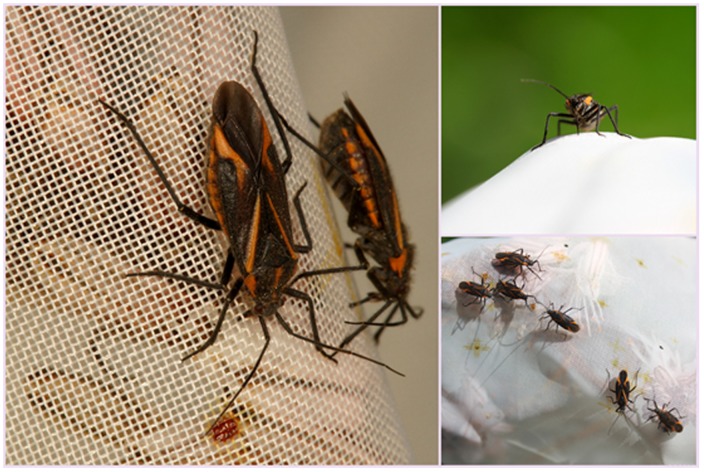
Adult mirid bugs (*Capsodes infuscatus*) on bagged flowers inserting their piercing-sucking mouthparts through the bag and into the flower. The orange marks on the covering bags are the bugs' waste.

The bagged treatment failed to exclude nectar consumers such as thrips (*Frankliniella* and *Thrips*) that were occasionally found in the bagged flowers in all sites. Moreover, we documented adult *C. infuscatus* bugs inserting their piercing-sucking mouthparts through the bag and into the flower and extracting their waste on the covering bag (Fig. 6).

## Discussion

Here we have demonstrated that bacterial communities in nectar collected from *Asphodelus aestivus* plants located along a steep climatic gradient differed significantly in composition across sites. The Bashan and Golan sites are the closest (∼10 km) and are also characterized by similar environmental conditions. However, according to cluster analysis, their community compositions as determined by the OTUs were less similar than were those at Bashan and Goral, which are distant geographically (∼200 km) and in terms of environmental factors (Fig. 4, Fig. 5 and Table 1). For example, the common bacterium *Gluconobacter* sp., which was previously shown to weaken plant–pollinator mutualism [32], was identified only at Golan. More broadly, these results also agree with the traditional hypothesis of niche assembly, since the first two CCA ordination axes explained 74% of the variance of the species–environment relationship and since some of the species (e.g. *Arthrobacter oryzae* and *Curtobacterium flaccumfaciens*), were found only at these two remote locations. To our knowledge, this is the first study on bacterial communities in floral nectar and its herbivores, conducted at sites located along a geographical and environmental gradient. This is also the first study of bacterial communities on mirid bugs.

Next generation sequencing techniques provide deep insights into the composition of microbial communities in ecological habitats. These strategies cover both culturable and unculturable bacterial species, but as sequences are short (∼300 bp.) the identification is accurate only up to the genera level [50]. Nevertheless, culturing is a common way to study bacterial communities in some environments such as food fermentation [51], [52] and nectar [29]–[30], [35], [53], where the fraction of culturable bacteria is higher than in other ecological habitats (e.g. marine environments) [28], [51], [52]. Using 16S rRNA 454-pyrosequencing, Fridman *et al*. [28] showed that 67–97% of the bacterial OTUs in nectar bacterial communities of three different plant species, belonged to the genus *Acinetobacter*. *Acinetobacter* isolates were also the dominant fraction when the culturable method was used [28]. At least eight different novel A*cinetobacter* species were identified (see Table 1 & Fig. 4 in ref. 28) and created an outgroup to the *Acinetobacter* type species, demonstrating that indeed, a significant fraction of the bacteria in nectar are culturable species. Still, it may be that not all the *Acinetobacter* OTUs were identified by culture [28]. We identified 161 representative isolates, which is a relatively large number compared with the number in other studies on culture-dependent bacterial communities in nectar [28], [30], [35] and on nectar-dwelling yeasts (e.g. [54], [55]). In particular, we note that Álvarez-Pérez & Herrera [30] found bacteria in 2.6% of the nectar samples of *A. aestivus*, while we isolated bacteria from all the examined samples. We attribute this to the different culturing methods, since those authors [30], used the rich medium trypticase soy agar (TSA) without sucrose, while we employed R2A agar (which contains far fewer proteins) supplemented with 30% sucrose which better mimics the nectars' nutritional environment. Culturable methods were also used for the bacterial communities on mirid bugs. It is known that insects harbor a significant fraction of symbionts which are unculturable [56]. However, insects' symbionts survive only inside their host and sometimes even only inside the hosts' cells so, it is very unlikely that they play a role in bacterial transfer from or into the floral nectar.

While a recent study found different bacterial communities in open and bagged flowers [31], we found no differences in bacterial communities between these two treatments. The similarity between open and bagged treatments in this study could be due to the ability of *C. infuscatus* bugs to penetrate the bag with their piercing-sucking mouthparts (Fig. 6) and due to the presence of a few thrips that we observed in some of the bagged flowers. We emphasize the ability of these species to circumvent the covering bag barrier, as the bagging method is considered a common practice in ecological studies. Furthermore, *Rosenbergiella nectarea* which was recently isolated and identified from nectar [28], [57], was also identified in the current study from nectar of *A. aestivus* (Table 2). Chanbusarakum and Ullman [58] isolated and identified the same bacterial species from western flower thrips (concluded from a 16S rRNA sequences alignment of *R. nectarea* and unidentified *Enterobacteriaceae* sp. from Chanbusarakum and Ullman [58]). Hence, this may indicate that thrips, which are tiny insects, feeding on pollen, are vectors of transmission of *Rosenbergiella nectarea* between floral nectar.

Our findings indicate that floral nectar supports higher bacterial species richness than do bugs (Fig. 3). However, this is probably due to bias introduced by the sucrose-rich medium used in the experiments to mimic the osmotic properties of the nectar. We conclude that both the *A. aestivus* nectar and the surface of *C. infuscatus* bugs support overlapping bacterial communities. The bug feeds on flowers [40], and in this study was documented doing so when visiting the flowers. Therefore, it is only reasonable to assume that bacteria are transferred to the nectar from the external surface of the bugs. Since the nectar is not consumed, strains found in the nectar are not likely to be found inside the bugs (although there is a possibility that bugs might defecate in flowers). Indeed, bacterial communities inside the bug differed from those found in the nectar. The similarities between mirid bugs and nectar bacterial communities imply that nectar bacterial dispersal is shaped not only by air-borne bacteria and nectar consumers as was found and discussed by Aizenberg-Gershtein et al. [31], but also by visiting vectors such as mirid bugs.

The differences we found in bacterial communities across sites, suggest that microbial model systems are useful for addressing macro-ecological questions. Such studies could have great impact, since micro- and macro-organisms seem to follow the same general ecological laws and patterns, albeit at different spatial and temporal scales [59]. Studying bacterial community composition could reveal the spatial and environmental processes that drive species assembly in communities.

## Supporting Information

Table S1 **List of bacterial isolates from nectar of** ***Asphodelus aestivus*** **in the four sites (Goral, Nadiv, Bashan and Golan) and within each treatment (open or bagged).** In the table, number of isolates and (in parentheses) coverage and percentage of the 16S rRNA gene similarities to the closest known species, respectively.
(PDF)

Table S2 **List of bacterial isolates from** ***Capsodes infuscatus*** **in the four sites (Goral, Nadiv, Bashan and Golan) and within each treatment (in or out).** In the table, number of isolates and (in parentheses) coverage and percentage of the 16S rRNA gene similarities to the closest known species, respectively.
(PDF)
