# Supplementary material for: The Role of Abiotic Environmental Conditions and Herbivory in Shaping Bacterial Community Composition in Floral Nectar
Source: PLoS One. 2014 Jun 12;9(6):e99107. doi: 10.1371/journal.pone.0099107 (PMC4055640; doi:10.1371/journal.pone.0099107)
Supplement: Table S1 — List of bacterial isolates from nectar of Asphodelus aestivus in the four sites (Goral, Nadiv, Bashan and Golan) and within each treatment (open or bagged). In the table, number of isolates and (in parentheses) coverage and percentage of the 16S rRNA gene similarities to the closest known species, respectively. (PDF) [file pone.0099107.s001.pdf]

Supplementary Tables

Table S1. List of bacterial isolates from nectar of *Asphodelus aestivus* in the four sites (Goral, Nadiv, Bashan and Golan) and within each treatment (open or bagged). In the table, number of isolates and (in parentheses) coverage and percentage of the 16S rRNA gene similarities to the closest known species, respectively.

**Class**	**Closest relative in GenBank database**	**Goral**	
		**Open**	**Bagged**
Actinobacteria	*Agromyces salentinus*		
	Arthrobacter humicola		
	Arthrobacter nitroguajacolicus		2 (809, 898; 99.5,99.8)
	Arthrobacter oryzae		1 (580; 99.4)
	Arthrobacter oxydans	1 (791; 100)	2 (703, 793; 99.9,100)
	Arthrobacter pascens		1 (721; 99.6)
	Arthrobacter phenanthrenivorans		
	Brevibacterium frigoritolerans		
	Curtobacterium flaccumfaciens	1 (745; 100)	
	Microbacterium foliorum		
Bacilli	*Bacillus aerophilus*		
	Bacillus anthracis		1 (875; 98.6)
	Bacillus aryabhattai	1 (785; 100)	1 (683; 100)
	Bacillus endophyticus		
	Bacillus flexus		
	Bacillus licheniformis	1 (869; 99.7)	
	Bacillus megaterium	1 (768; 99.7)	
	Bacillus mojavensis	1 (862; 100)	
	Bacillus nealsonii		1 (700; 99.0)
	Bacillus niacini		1 (718; 99.8)
	Bacillus safensis	3 (770, 791, 806; 99.8-100)	
	Bacillus simplex	2 (764, 794; 100)	1 (752; 100)
	Bacillus sonorensis	1 (877; 99.0)	
	Bacillus subtilis subsp. inaquosorum	1 (769; 99.9)	
	Bacillus tequilensis	7 (701, 782, 782, 782 786, 812, 832; 99.9)	
	Brevibacillus agri		
	Fictibacillus nanhaiensis		1 (778; 99.5)
	Leuconostoc holzapfelii		
	Lysinibacillus sinduriensis		
	Scopulibacillus darangshiensis	1 (799; 95.0)	
	Staphylococcus arlettae	1 (829; 100)	
	Staphylococcus cohnii subsp. cohnii		
	Staphylococcus cohnii subsp. urealyticus		3 (710, 718, 782; 100)
	Staphylococcus epidermidis		
	Staphylococcus hominis subsp. hominis		
	Staphylococcus warneri		1 (709; 100)
	Terribacillus saccharophilus		
Alphaproteobacteria	*Gluconobacter kondonii*		
	Gluconobacter morbifer		
	Gluconobacter sphaericus		
	Neokomagataea tanensis		
Gammaproteobacteria	*Acinetobacter boissieri*		
	Acinetobacter nectaris		
	Erwinia persicina		
	Erwinia toletana	2 (779, 865; 99.7)	1 (773; 99.7)
	Flavimonas oryzihabitans	1 (843; 99.2)	
	Lonsdalea quercina		
	Pantoea eucalypti		
	Pseudomonas azotoformans		
	Pseudomonas baetica		
	Pseudomonas cedrina subsp. fulgida		
	Pseudomonas congelans		
	Pseudomonas graminis		1 (870; 99.4)
	Pseudomonas koreensis		1 (741; 100)
	Pseudomonas lini		
	Pseudomonas lutea		1 (812; 100)
	Pseudomonas mohnii		1 (598; 99.6)
	Pseudomonas syringae		
	Pseudomonas viridiflava		
	Rosenbergiella nectarea	1 (867; 99.5)	
	**Total**	**26**	**21**

Class	Closest relative in GenBank database	Nadiv	
		Open	Bagged
Actinobacteria	*Agromyces salentinus*		
	Arthrobacter humicola		
	Arthrobacter nitroguajacolicus		
	Arthrobacter oryzae		
	Arthrobacter oxydans		
	Arthrobacter pascens		
	Arthrobacter phenanthrenivorans		
	Brevibacterium frigoritolerans		
	Curtobacterium flaccumfaciens		
	Microbacterium foliorum		
Bacilli	*Bacillus aerophilus*		
	Bacillus anthracis		
	Bacillus aryabhattai		
	Bacillus endophyticus		
	Bacillus flexus		
	Bacillus licheniformis		
	Bacillus megaterium		
	Bacillus mojavensis		
	Bacillus nealsonii		1 (875; 99.2)
	Bacillus niacini		
	Bacillus safensis		
	Bacillus simplex		
	Bacillus sonorensis		
	Bacillus subtilis subsp. inaquosorum		1 (787; 99.9)
	Bacillus tequilensis	2 (737, 764; 99.9,100)	2 (716, 760; 99.9)
	Brevibacillus agri		
	Fictibacillus nanhaiensis		
	Leuconostoc holzapfelii		
	Lysinibacillus sinduriensis		
	Scopulibacillus darangshiensis		
	Staphylococcus arlettae		
	Staphylococcus cohnii subsp. cohnii		
	Staphylococcus cohnii subsp. urealyticus		
	Staphylococcus epidermidis		
	Staphylococcus hominis subsp. hominis		
	Staphylococcus warneri		
	Terribacillus saccharophilus		
Alphaproteobacteria	*Gluconobacter kondonii*		
	Gluconobacter morbifer		
	Gluconobacter sphaericus		
	Neokomagataea tanensis	2 (656, 810; 100)	
Gammaproteobacteria	*Acinetobacter boissieri*	1 (754; 100)	
	Acinetobacter nectaris	4 (773, 790, 807, 905; 99.8-100)	
	Erwinia persicina		
	Erwinia toletana		
	Flavimonas oryzihabitans		
	Lonsdalea quercina		
	Pantoea eucalypti		
	Pseudomonas azotoformans		
	Pseudomonas baetica		
	Pseudomonas cedrina subsp. fulgida		
	Pseudomonas congelans		
	Pseudomonas graminis		
	Pseudomonas koreensis		
	Pseudomonas lini		
	Pseudomonas lutea		
	Pseudomonas mohnii		
	Pseudomonas syringae		
	Pseudomonas viridiflava	1 (848; 100)	
	Rosenbergiella nectarea	1 (855; 98.9)	
	**Total**	**11**	**4**

Class	Closest relative in GenBank database	Bashan	
		Open	Bagged
Actinobacteria	*Agromyces salentinus*		1 (709; 99.9)
	Arthrobacter humicola		
	Arthrobacter nitroguajacolicus		
	Arthrobacter oryzae	1 (723; 99.9)	1 (720; 99.9)
	Arthrobacter oxydans	2 (713, 542; 100)	
	Arthrobacter pascens		1 (782; 99.7)
	Arthrobacter phenanthrenivorans		1 (716; 98.7)
	Brevibacterium frigoritolerans		2 (735, 740; 100)
	Curtobacterium flaccumfaciens	2 (708, 765; 99.9-100)	
	Microbacterium foliorum		1 (710; 99.3)
Bacilli	*Bacillus aerophilus*	1 (778; 100)	1 (710; 100)
	Bacillus anthracis		
	Bacillus aryabhattai		
	Bacillus endophyticus	1 (739; 99.7)	
	Bacillus flexus	1 (721; 100)	
	Bacillus licheniformis		
	Bacillus megaterium		1 (698; 99.7)
	Bacillus mojavensis		
	Bacillus nealsonii		
	Bacillus niacini		
	Bacillus safensis		
	Bacillus simplex	1 (730; 100)	1 (734; 100)
	Bacillus sonorensis		
	Bacillus subtilis subsp. inaquosorum		
	Bacillus tequilensis		
	Brevibacillus agri		
	Fictibacillus nanhaiensis		
	Leuconostoc holzapfelii	1 (759; 99.9)	
	Lysinibacillus sinduriensis		1 (740; 99.1)
	Scopulibacillus darangshiensis		
	Staphylococcus arlettae		
	Staphylococcus cohnii subsp. cohnii	1 (749; 100)	
	Staphylococcus cohnii subsp. urealyticus		
	Staphylococcus epidermidis	1(780; 100)	
	Staphylococcus hominis subsp. hominis		2 (683, 710; 99.7,99.9)
	Staphylococcus warneri	1 (751; 100)	
	Terribacillus saccharophilus		1 (596; 100)
Alphaproteobacteria	*Gluconobacter kondonii*		
	Gluconobacter morbifer		
	Gluconobacter sphaericus		
	Neokomagataea tanensis		
Gammaproteobacteria	*Acinetobacter boissieri*		
	Acinetobacter nectaris		
	Erwinia persicina		
	Erwinia toletana		
	Flavimonas oryzihabitans		
	Lonsdalea quercina		
	Pantoea eucalypti		
	Pseudomonas azotoformans		
	Pseudomonas baetica	1 (819; 99.3)	
	Pseudomonas cedrina subsp. fulgida		2 (719, 730; 100)
	Pseudomonas congelans		
	Pseudomonas graminis		
	Pseudomonas koreensis		
	Pseudomonas lini	10 (619, 705, 714, 763, 782, 790, 827, 829, 831, 844; 99.4-100)	
	Pseudomonas lutea		
	Pseudomonas mohnii		
	Pseudomonas syringae	7 (503, 711, 827, 861, 862, 902, 910; 99.2-99.8)	
	Pseudomonas viridiflava		
	Rosenbergiella nectarea		
	**Total**	**31**	**16**

Class	Closest relative in GenBank database	Golan	
		Open	Bagged
Actinobacteria	*Agromyces salentinus*		
	Arthrobacter humicola	2 (795, 873; 99.2,99.3)	3 (714, 811, 839; 99.3-99.4)
	Arthrobacter nitroguajacolicus		
	Arthrobacter oryzae		
	Arthrobacter oxydans		
	Arthrobacter pascens	1 (791; 99.5)	1 (717; 99.4)
	Arthrobacter phenanthrenivorans		1 (771; 99.5)
	Brevibacterium frigoritolerans		
	Curtobacterium flaccumfaciens		5 (677, 698, 756, 793, 848; 99.0-100)
	Microbacterium foliorum		
Bacilli	*Bacillus aerophilus*	1 (892; 100)	
	Bacillus anthracis		
	Bacillus aryabhattai	5 (785, 819, 834,859, 863; 99.8-100)	1 (855; 99.9)
	Bacillus endophyticus		
	Bacillus flexus		
	Bacillus licheniformis		
	Bacillus megaterium		
	Bacillus mojavensis		
	Bacillus nealsonii		
	Bacillus niacini		
	Bacillus safensis	1 (722; 99.9)	
	Bacillus simplex	4 (824, 825, 858, 874; 100)	6 (534, 818, 827, 829, 836, 898; 99.6-100)
	Bacillus sonorensis		
	Bacillus subtilis subsp. inaquosorum		
	Bacillus tequilensis		1 (877; 99.9)
	Brevibacillus agri	2 (519, 842; 98.7; 99.4)	1 (800; 99.4)
	Fictibacillus nanhaiensis		
	Leuconostoc holzapfelii		
	Lysinibacillus sinduriensis		
	Scopulibacillus darangshiensis		
	Staphylococcus arlettae		
	Staphylococcus cohnii subsp. cohnii		
	Staphylococcus cohnii subsp. urealyticus		
	Staphylococcus epidermidis		
	Staphylococcus hominis subsp. hominis		
	Staphylococcus warneri	1 (844; 100)	
	Terribacillus saccharophilus		1 (596; 100)
Alphaproteobacteria	*Gluconobacter kondonii*	4 (757, 803, 847, 858; 98.0-98.4)	
	Gluconobacter morbifer	2 (753, 901; 97.2-98.0)	
	Gluconobacter sphaericus	1 (863; 98.4)	
	Neokomagataea tanensis		
Gammaproteobacteria	*Acinetobacter boissieri*		
	Acinetobacter nectaris		
	Erwinia persicina	2 (837, 866; 99.7-99.8)	
	Erwinia toletana		
	Flavimonas oryzihabitans		
	Lonsdalea quercina	1 (866; 99.9)	
	Pantoea eucalypti	1 (830; 99.8)	
	Pseudomonas azotoformans	2 (811, 861; 99.9)	
	Pseudomonas baetica		
	Pseudomonas cedrina subsp. fulgida		
	Pseudomonas congelans	1 (822; 100)	
	Pseudomonas graminis		
	Pseudomonas koreensis		
	Pseudomonas lini		
	Pseudomonas lutea		
	Pseudomonas mohnii		
	Pseudomonas syringae		
	Pseudomonas viridiflava	1 (826; 99.6)	
	Rosenbergiella nectarea		
	**Total**	**32**	**20**
