# Supplementary material for: The Role of Abiotic Environmental Conditions and Herbivory in Shaping Bacterial Community Composition in Floral Nectar
Source: PLoS One. 2014 Jun 12;9(6):e99107. doi: 10.1371/journal.pone.0099107 (PMC4055640; doi:10.1371/journal.pone.0099107)
Supplement: Table S2 — List of bacterial isolates from Capsodes infuscatus in the four sites (Goral, Nadiv, Bashan and Golan) and within each treatment (in or out). In the table, number of isolates and (in parentheses) coverage and percentage of the 16S rRNA gene similarities to the closest known species, respectively. (PDF) [file pone.0099107.s002.pdf]

Supplementary Tables

Table S2. List of bacterial isolates from *Capsodes infuscatus* in the four sites (Goral, Nadiv, Bashan and Golan) and within each treatment (in or out). In the table, number of isolates and (in parentheses) coverage and percentage of the 16S rRNA gene similarities to the closest known species, respectively.

**Class**	**Closest relative in GenBank database**	**Goral**	
		**In**	**Out**
Actinobacteria	*Arthrobacter oxydans*		
	Curtobacterium flaccumfaciens		
	Microbacterium foliorum	2 (521, 739; 99.2-99.3)	
Bacilli	*Bacillus aerophilus*		
	Bacillus anthracis		
	Bacillus aryabhattai		1 (718; 100)
	Bacillus cereus		
	Bacillus endophyticus		
	Bacillus flexus		
	Bacillus licheniformis		
	Bacillus nealsonii		
	Bacillus safensis		
	Bacillus simplex		
	Bacillus subtilis subsp. inaquosorum	1 (584; 100)	
	Bacillus tequilensis	2 (841, 872; 100)	2 (784, 786; 99.9)
	Brevibacillus agri		
	Staphylococcus saprphyticus		
	Staphylococcus warneri		
Alphaproteobacteria	*Gluconobacter morbifer*		
Gammaproteobacteria	*Acinetobacter schindleri*	1 (799; 99.9)	
	Erwinia persicina		
	Erwinia toletana		
	Flavimonas oryzihabitans		
	Pantoea agglomerans		

Class	Closest relative in GenBank database	Goral	
		In	Out
Gammaproteobacteria	*Pantoea brenneri*		
	Pantoea conspicua		
	Pseudomonas costantinii		
	Pseudomonas orientalis		
	Pseudomonas plecoglossicida		
	Yersinia kristensenii		
	**Total**	**6**	**3**

Class	Closest relative in GenBank database	Nadiv	
		In	Out
Actinobacteria	*Arthrobacter oxydans*		
	Curtobacterium flaccumfaciens	1 (848; 100)	
	Microbacterium foliorum		
Bacilli	*Bacillus aerophilus*		1 (734, 100)
	Bacillus anthracis		
	Bacillus aryabhattai		
	Bacillus cereus		
	Bacillus endophyticus		
	Bacillus flexus		
	Bacillus licheniformis		1 (889; 99.8)
	Bacillus nealsonii		1 (856; 99.3)
	Bacillus safensis		
	Bacillus simplex		
	Bacillus subtilis subsp. inaquosorum		
	Bacillus tequilensis	1 (669; 100)	1 (784; 99.9)
	Brevibacillus agri		
	Staphylococcus saprphyticus		
	Staphylococcus warneri	1 (983; 99.0)	
Alphaproteobacteria	*Gluconobacter morbifer*		
Gammaproteobacteria	*Acinetobacter schindleri*		
	Erwinia persicina	1 (607; 99.2)	3 (723-757; 99.3-99.9)
	Erwinia toletana	2 (868, 873; 96.1)	3 (685-846; 94.9-95.7)
	Flavimonas oryzihabitans		4 (812-833; 99.1)
	Pantoea agglomerans	3(673-831; 99.4-99.7)	4 (676-863; 99.4-99.8)
	Pantoea brenneri	7 (778-888; 99.6-100)	3 (704-795; 99.7-100)
	Pantoea conspicua	2 (761, 879; 95.0-95.4)	1 (789; 94.9)
	Pseudomonas costantinii		1 (779; 99.4)
	Pseudomonas orientalis		
	Pseudomonas plecoglossicida	1 (745; 99.3)	
	Yersinia kristensenii		
	**Total**	**19**	**23**

Class	Closest relative in GenBank database	Bashan	Golan	
		Out	In	Out
Actinobacteria	*Arthrobacter oxydans*		1 (897; 99.3)	
	Curtobacterium flaccumfaciens			1 (869; 100)
	Microbacterium foliorum			
Bacilli	*Bacillus aerophilus*		26 (679-929; 99.6-100)	
	Bacillus anthracis		2 (818, 875; 100)	
	Bacillus aryabhattai		1 (879; 100)	
	Bacillus cereus		3 (739-845; 99.9)	
	Bacillus endophyticus	2 (719, 832; 99.7)		
	Bacillus flexus	1 (878; 100)		
	Bacillus licheniformis			1 (769; 96.7)
	Bacillus nealsonii			
	Bacillus safensis	1 (499; 100)		
	Bacillus simplex			2 (889, 899; 100)
	Bacillus subtilis subsp. inaquosorum			
	Bacillus tequilensis			
	Brevibacillus agri	1 (838; 99.4)	1 (839; 99.6)	
	Staphylococcus saprphyticus		1 (869; 99.9)	2 (866, 891; 99.9)
	Staphylococcus warneri			
Alphaproteobacteria	*Gluconobacter morbifer*		1 (834; 98.1)	
Gammaproteobacteria	*Acinetobacter schindleri*			
	Erwinia persicina			
	Erwinia toletana			
	Flavimonas oryzihabitans			
	Pantoea agglomerans			
	Pantoea brenneri			
	Pantoea conspicua			
	Pseudomonas costantinii			
	Pseudomonas orientalis		1 (861; 100)	4 (792-861; 99.8-100)
	Pseudomonas plecoglossicida			
	Yersinia kristensenii		10 (868-914; 98.8-99.3)	2 (832, 865; 99.3)
	**Total**	**5**	**47**	**12**
